# The Prevalence of Antibiotic Tolerance in *Neisseria gonorrhoeae* Varies by Anatomical Site

**DOI:** 10.3390/pathogens13070538

**Published:** 2024-06-26

**Authors:** Margaux Balduck, Akim Strikker, Zina Gestels, Saïd Abdellati, Dorien Van den Bossche, Irith De Baetselier, Chris Kenyon, Sheeba Santhini Manoharan-Basil

**Affiliations:** 1 STI Unit, Department of Clinical Sciences, Institute of Tropical Medicine, 2000 Antwerp, Belgium; margaux.balduck@ki.se (M.B.); akimstrikker98@gmail.com (A.S.); ckenyon@itg.be (C.K.); sbasil@itg.be (S.S.M.-B.); 2 Clinical and Reference Laboratory, Department of Clinical Sciences, Institute of Tropical Medicine, 2000 Antwerp, Belgium; sabdellati@itg.be (S.A.); dvandenbossche@itg.be (D.V.d.B.); idebaetselier@itg.be (I.D.B.); 3 Division of Infectious Diseases and HIV Medicine, University of Cape Town, Cape Town 7700, South Africa

**Keywords:** *Neisseria gonorrhoeae*, tolerance, antimicrobial resistance, anorectal infection, urogenital infection, azithromycin, ceftriaxone, ciprofloxacin

## Abstract

Background: Tolerance enables bacteria to survive intermittent antibiotic exposure without an increase in antimicrobial susceptibility. In this study, we investigated the presence of tolerance to three antimicrobials, ceftriaxone, azithromycin and ciprofloxacin, in clinical isolates and the WHO (World Health Organization) reference panel of *Neisseria gonorrhoeae*. Methods: We used the modified tolerance disk (TD test) to assess for tolerance to ceftriaxone, azithromycin and ciprofloxacin in 14 WHO reference strains and 62 *N. gonorrhoeae* clinical isolates—evenly divided between anorectal and urogenital infections. The isolates underwent a three-step incubation process wherein the isolates were exposed to an antibiotic disk for 20 h of incubation (Step I), followed by the replacement of the antibiotic disk with a nutrient disk for overnight incubation (Step II) and additional overnight incubation with extra nutrients (Step III). Results: A total of 4 of the 62 clinical anorectal isolates and none of the urogenital isolates exhibited tolerance to azithromycin (*p* = 0.033). Tolerance to ceftriaxone and ciprofloxacin was observed in eight and four isolates, respectively, with no difference between infection sites. Tolerance was also detected in 8 (K, M, N, O, P, U, V, W) out of the 14 WHO reference strains, with varying patterns of tolerance to ceftriaxone (*n* = 8), ciprofloxacin (*n* = 2) and azithromycin (*n* = 1). Conclusions: This study identified ceftriaxone, azithromycin and ciprofloxacin tolerance in clinical and WHO reference *N. gonorrhoeae* isolates. Azithromycin tolerance was more common in anorectal than urogenital infections.

## 1. Introduction

Tolerance is defined as the ability of bacteria to survive transient exposure to high bactericidal concentrations of antibiotics by slowing their metabolism through an extension of the lag phase without a change in the minimum inhibitory concentration (MIC) [1,2,3]. Antibiotic tolerance has been shown to play an important role in the emergence of antimicrobial resistance (AMR) [2,4]. Notably, in *Escherichia coli*, tolerance has been shown to accelerate the development of AMR and has been implicated in treatment failure [5]. Recent studies have validated the use of the cheap and easy-to-perform tolerance disk (TD) test for detecting tolerance in clinical isolates of bacteria such as *Staphylococcus aureus* and *E. coli* [6,7].

*N. gonorrhoeae* has developed resistance to multiple classes of antibiotics, including ceftriaxone (CRO), the recommended treatment for gonorrhoea. Due to the increase in antimicrobial resistance (AMR), there is a real possibility that *N. gonorrhoeae* may become untreatable [8,9,10]. Previously, we demonstrated that tolerance to ceftriaxone (CRO) could be induced in *N. gonorrhoeae* by intermittent exposure to a high concentration of CRO followed by the detection of tolerance in *N. gonorrhoeae* using the modified TD test [11]. Furthermore, using the modified TD test, we detected CRO tolerance in clinical isolates of *N. gonorrhoeae* [11]. In addition, our study found that *N. gonorrhoeae* isolates from the anorectum were more likely to be CRO-tolerant than isolates from urogenital sites. However, the limited number of samples assessed was too small to warrant assessing if the difference was statistically significant [11]. Moreover, this study only evaluated tolerance to a single antimicrobial [11]. Previous studies have found important pheno- and genotypic differences between *N. gonorrhoeae* isolated from different anatomical sites [12,13].

These findings led to the current study, where we aimed to assess if tolerance to three antimicrobials, azithromycin (AZM), ceftriaxone (CRO) and ciprofloxacin (CIP), could be detected in clinical isolates and the WHO reference panel of *N. gonorrhoeae* using the previously established modified TD test. Azithromycin is typically classified as a bacteriostatic antimicrobial. However, at higher doses (>2 times the MIC), it exerts a bactericidal effect against *N. gonorrhoeae* [14]. In addition, we aimed to assess if the prevalence of tolerance to each antimicrobial differed between urogenital and anorectal infection sites. Finally, we aimed to assess if AMR emerged more rapidly in tolerant isolates than non-tolerant ones.

## 2. Materials and Methods 

### 2.1. Bacterial Strains

(i) WHO Reference Panel

Fourteen gonococcal WHO reference strains were used in this study (F, G, K, L, M, N, O, P, U, V, W, X, Y, Z ) [15].

(ii) Clinical Isolates

*N. gonorrhoeae* clinical isolates (*n* = 66) from 66 different individuals in the Belgian National Reference Centre of STIs (2023) were selected for this study. The isolates were equally divided between anorectal (*n* = 33) and urogenital (*n* = 33) infection sites. The isolates were randomly selected by an independent researcher who was instructed to select 33 anorectal and 33 urogenital isolates received by the Belgian National Reference Centre of STIs in 2023.

The MICs of the three antimicrobials (AZM, CRO and CIP) were determined using the E-test method, following the manufacturer’s instructions (BioMérieux, France) on gonococcal (GC) agar plates [3.6% BD Difco^TM^ GC Medium Base (Fisher Scientific, Waltham, MA, USA) supplemented with 1% IsoVitalex (BD)] (Supplementary Table S1). The isolates were revived from skim milk with 20% glycerol, stored at −80 °C. They were subcultured and incubated twice on BD^TM^ Columbia Agar with 5% Sheep Blood at 5.5% CO_2_ atmosphere and 36 °C.

### 2.2. Tolerance Detection 

**Figure 1:**
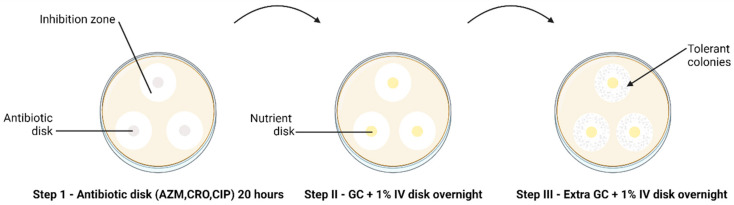
The TD test of clinical *N. gonorrhoeae* isolates (*n* = 66) with 0.75 µg AZM, 0.064 µg CRO and 0.032 µg CIP 6 mm disks, performed in triplicate. The arrow indicates the presence of tolerant colonies after Step III of the TD test. Discs containing antibiotics are shown in grey and discs containing nutrient are shown in yellow.

The modified TD test was carried out according to Balduck et al., 2022 [11]. This test comprises a three-step incubation process. In brief, the direct colony suspension method was used to prepare the inoculum [16], wherein a small quantity of inoculum from an overnight subculture on chocolate agar was suspended in phosphate buffer saline (PBS). The turbidity of the suspension was adjusted to 0.5–1.0 to the McFarland (McF) standard and inoculated on BD^TM^ GC agar plates. Three disks with the respective antibiotics (AZM 0.75 µg/mL, CRO 0.064 µg/mL, CIP 0.032 µg/mL) were placed on each plate inoculated with the strains. Antibiotic disks were created by soaking 6 mm blank disks (Merck Life Science, Darmstadt, Germany) in 25 µL of the required antibiotic, and the antibiotic concentration was calculated to fall below the MIC. After approximately 20 to 24 h of incubation at 36° C and 6.0% CO_2_ (Step I TD test), the antibiotic disks were removed and replaced by 6 mm blank disks containing 25 µL gonococcal broth (distilled water supplemented with 15 g/L bacto protease peptone n^o^ 3, 1 g/L soluble starch, 4 g/L K_2_HPO_4_ (174.18 g/mol), 1 g/L KH_2_PO_4_ (136.08 g/mol), 5 g/L NaCl (58.44 g/mol) and 1% BD BBL^TM^ IsoVitaleX). The plates were again incubated overnight (Step II TD test). This was followed by adding 10 µL of GC medium onto the same nutrient disk. The plates were incubated for another night (Step III TD test) (Figure 1).

All plates were analysed for tolerance by three independent researchers and were photographed. The TD test was duplicated and triplicated for the WHO reference and clinical *N. gonorrhoeae* isolates, respectively. If tolerant colonies were found, they were harvested and grown on BD^TM^ GC chocolate agar, incubated for 48 h and stored in 20% glycerol skim milk at −80 °C. 

### 2.3. Antimicrobial Susceptibility Testing 

Following the TD tests, the MIC of tolerant colonies was determined using the E-test method (range AZM: 0.016 µg/mL–256 µg/mL CRO: 0.016 µg/mL–256 µg/mL, CIP: 0.002–32 µg/mL) (BioMérieux, Marcy-l’Étoile, France), according to the manufacturer’s instructions on GC agar plates [17].

### 2.4. Induction of Resistance to Ciprofloxacin in Ceftriaxone-Tolerant Colonies

In our previous study, we found that the CRO MICs of the CRO-tolerant isolates of *N. gonorrhoeae* did not increase faster than those of non-tolerant isolates [11]. Rather, we found that 8 days of exposure to crossover CRO E-tests did not increase the CRO MIC. This is compatible with other studies that found that the in vitro induction of CRO takes weeks to months and does not select for mutations commonly detected in clinical isolates with clinical resistance. In contrast, in vitro CIP exposure leads to a rapid increase in CIP MICs, and the emergent mutations are clinically relevant [18]. These findings provided the motivation for the current study, in which we assessed whether CRO-tolerant isolates accelerate the increase in ciprofloxacin MICs under ciprofloxacin selection pressure. To conduct this, we assessed the daily change in the CIP MIC in 4 randomly selected CRO-tolerant isolates and 4 randomly selected non-tolerant isolates. All these isolates were from the anorectum.

The crossover E-test method of Raisman et al. was followed [19]. Overnight cultures were used to produce bacterial suspensions (0.5–1.0 McF) in phosphate buffer saline (PBS) from colonies that were stored at −80 °C. These were then inoculated on a BD^TM^ GC agar plate. A ciprofloxacin gradient MIC strip/E-test (BioMérieux, Marcy-l’Étoile, France) was then placed on the plates to apply a selective pressure. These were then incubated overnight at 36 °C and 5.5% CO_2_. The following day, the overnight growth was collected from the zone of inhibition and a 1 cm margin around the zone of inhibition. This culture was suspended in PBS and re-inoculated on a GC agar plate with a CIP E-test. This was repeated every day for 7 consecutive days. These experiments were conducted in triplicate.

### 2.5. Statistical Analysis

Fisher’s exact test implemented in XLSTAT (https://www.xlstat.com/enersion accessed on 14 May 2024, 28.0; IBM Corporation, Armonk, NY, USA) was used to determine the association between the prevalence of tolerance/non-tolerance isolates and infection sites (anorectal, urogenital) for each antibiotic (AZM, CRO, CIP). Three replicates per clinical isolate were available. If all three replicates of the clinical isolates displayed tolerant colonies, the isolate was categorized as ‘tolerant’. If only one or two out of the three replicates exhibited tolerant colonies, they were classified as ‘possible-tolerant’. A *p*-value lower than 0.05 was considered statistically significant. Differences in MICs between groups were tested using the Mann–Whitney test. Mixed-effects linear regression was used to assess if CRO tolerance was associated with increases in the CIP MIC, with ‘delta_ciprofloxacin_MIC’ as the dependent variable (the change in the CIP MIC from the previous day). The predictor variables included time and tolerance with random intercepts specified for each strain.

The model was specified as follows:

‘xtmixed MIC Time Tolerance || Strain:’

## 3. Results

Of the 66 clinical isolates, 1 urogenital and 3 anorectal isolates were lost during the TD test due to contamination, resulting in 30 anorectal and 32 urogenital clinical isolates. The following isolates were lost: one replicate from the WHO reference panel (WHO V) and four clinical isolates. Urethral isolates had lower azithromycin MICs (median 0.125 mg/L; IQR 0.06–0.38) than anorectal isolates (median 1 mg/L; IQR 0.38–1.5; *p* < 0.0001; Table S5). Likewise, urethral isolates had lower ciprofloxacin MICs (median 0.002 mg/L; IQR 0.002–0.003) than anorectal isolates (median 0.020 mg/L; IQR 0.06–1.5; *p* < 0.0001).

### 3.1. Detection of Tolerance and Heterotolerance in WHO Reference Strains

Out of the fourteen isolates, tolerance to AZM, CIP and CRO was detected in one (WHO Z), one (WHO P) and seven WHO isolates (K, M, N, O, P, U and W), respectively (Supplementary Table S1, Table 1). The TD tests were performed in duplicate, which resulted in the identification of tolerance in one of two replicates for CIP and CRO in WHO-U (Supplementary Table S1). Pictures of the TD tests are available upon request.

### 3.2. Differences in Azithromycin Tolerance across Infection Sites 

**Figure 2:**
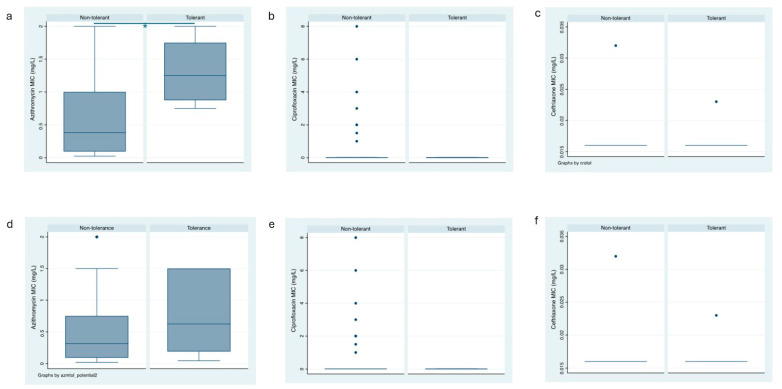
Box plots of the azithromycin, ceftriaxone and ciprofloxacin MICs of the tolerant and non-tolerant isolates (**a**–**c**) and the potentially tolerant and non-tolerant isolates (**d**–**f**); box represents median [line] and interquartile ranges, whiskers the 95% CI).

A total of 4 (6.5%) of the 62 clinical *N. gonorrhoeae* isolates exhibited tolerance to AZM (Figure 2a). Among the 30 anorectal clinical isolates, 4 (12.5%) showed tolerance to AZM, whereas among the 32 urogenital clinical isolates, none exhibited AZM tolerance (*p* = 0.033, Table 2). In a similar vein, the prevalence of possible AZM tolerance was higher in anorectal isolates [10/30 (33%]) than in urethral isolates (1/32 [3%]; *p* = 0.004, Table 2, Figure 2d). 

### 3.3. No Difference in Tolerance to Ceftriaxone and Ciprofloxacin between the Infection Sites

Tolerance to CIP was detected in 4 (6.5%) of the 62 clinical *N. gonorrhoeae* isolates (Figure 2b). A total of 2 isolates each from the 30 anorectal (6.7%) and the 32 urogenital (6.3%) clinical isolates showed tolerance to CIP, respectively (Table 2, Supplementary Table S2). Possible CIP tolerance was detected in 25 (40.3%) of the 62 clinical *N. gonorrhoeae* isolates—4/26 (13.3%) anorectal isolates and 7/25 (21.9%) exhibited AZM tolerance (*p* = 0.370, Table 2, Figure 2e). 

The prevalence of tolerance to CRO in the clinical isolates of *N. gonorrhoeae* was 8 (12.9%) out of 62 (Figure 2c). Tolerance to CRO was similar in isolates from anorectal infection and urogenital sites (6/24 [20%] and 2/30 (6.3%), respectively; *p* = 0.107, Table 2). The prevalence of possible tolerance to CRO was higher in anorectal infections than in urogenital infection sites (10/30 [33%] and 2/32 [6.3%], respectively; *p* = 0.007, Table 2, Figure 2f). The detected tolerant colonies did not have an increase in the MIC compared to the baseline isolates (Supplementary Table S3). 

### 3.4. Association between Ciprofloxacin and Ceftriaxone Tolerance

The prevalence of ceftriaxone tolerance in the clinical isolates was not associated with the prevalence of azithromycin or ciprofloxacin tolerance (Table 3). There was, however, a trend in this direction for ciprofloxacin–ceftriaxone (*p* = 0.077). There was no association between ciprofloxacin and azithromycin tolerance.

### 3.5. Induction of Resistance to Ciprofloxacin in CRO-Tolerant Isolates

**Figure 3:**
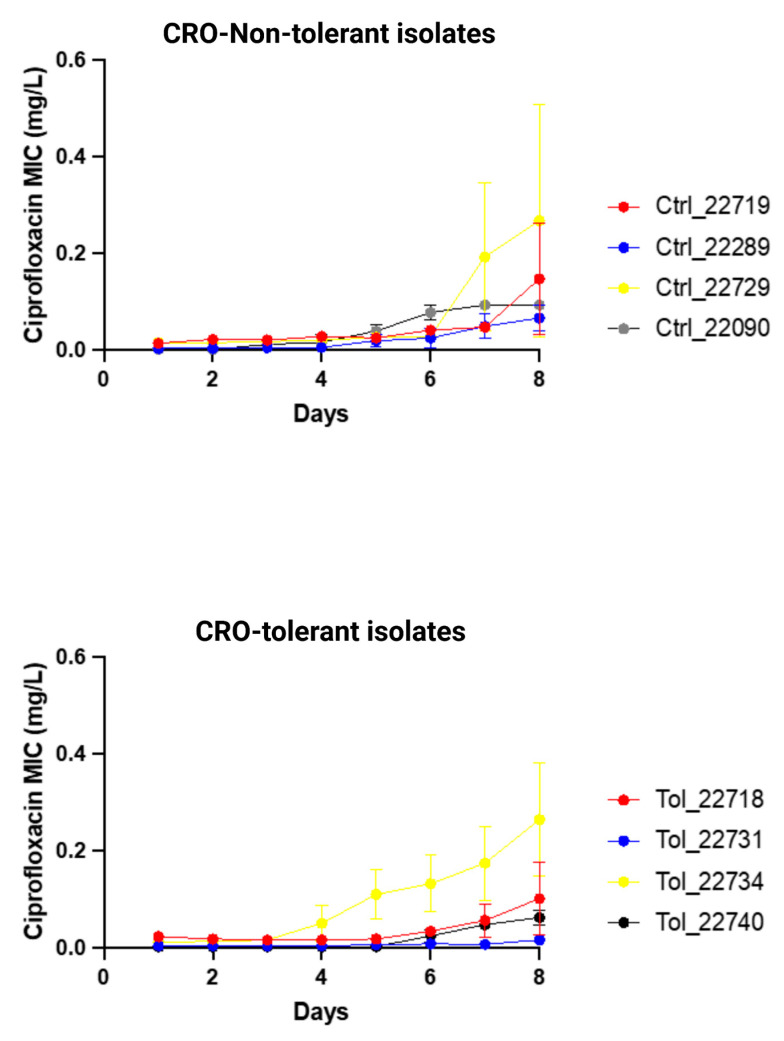
Changes in ciprofloxacin MIC (mg/L) of ceftriaxone-tolerant and -non-tolerant isolates of *N. gonorrhoeae* during exposure to ciprofloxacin selection pressure (means and standard error of mean shown of three replicates).

Ciprofloxacin resistance (MIC > 0.06 mg/L) emerged in three out of four CRO-tolerant clinical isolates (*n* = 4) and in all non-tolerant clinical isolates (*n* = 4; Figure 3; Supplementary Table S4). There was no significant difference in the increase in ciprofloxacin MICs between tolerant and non-tolerant isolates (Figure 3; Table 4; Supplementary Table S4). 

## 4. Discussion 

We previously established that tolerance to CRO could be detected in *N. gonorrhoeae* clinical isolates, but this was limited to a small sample size [11]. The current study confirms the previous findings using a larger sample size, 14 of the WHO reference strains and using three antibiotics to detect tolerance. In the clinical *N. gonorrhoeae* isolates, we found that the prevalence of tolerance to AZM (but not CRO or CIP) was higher in anorectal clinical isolates than in urogenital clinical isolates. The prevalence of possible CRO tolerance was also higher in the anorectal isolates.

**Figure 4:**
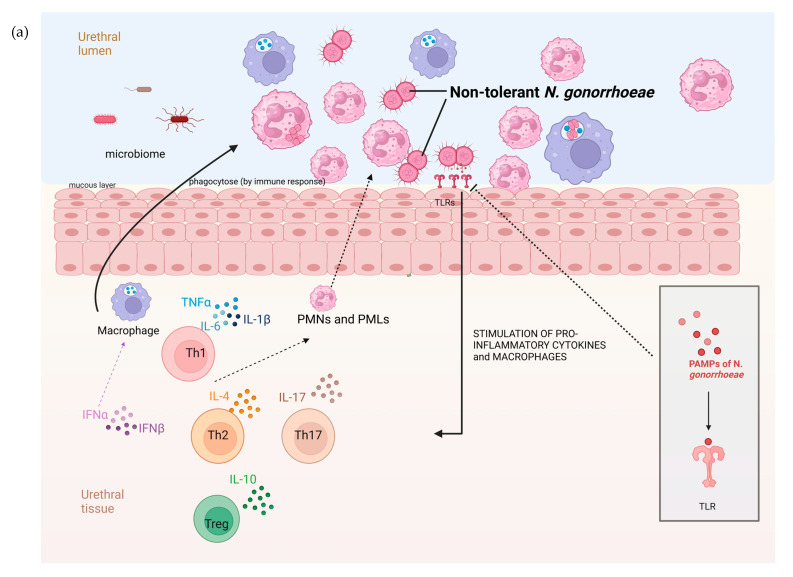

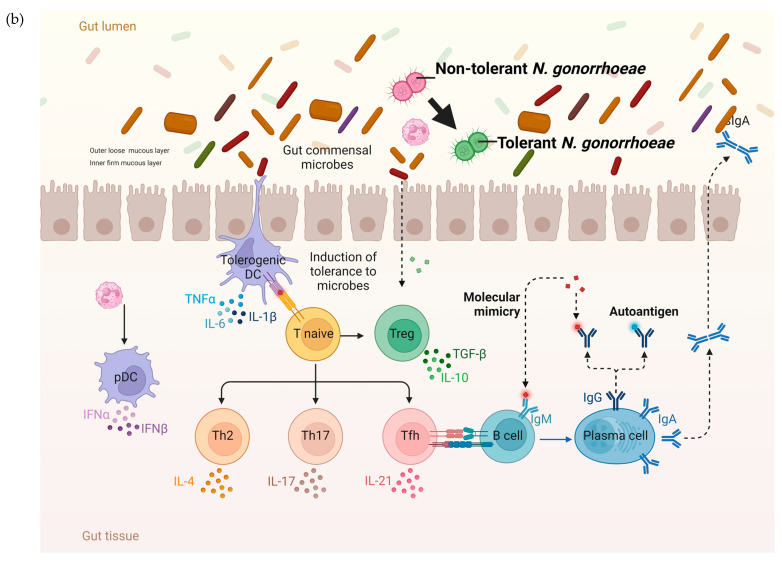
The hypothesized pathophysiology of the higher prevalence of *N. gonorrhoeae* tolerance in the rectum than the urethra. (**a**) The relative paucity of commensal microbes and related pronounced immune response in the mucosa of the urogenital tract result in a decreased probability of tolerance developing in *N. gonorrhoeae*. (**b**) In contrast, the abundance of microbes in the GIT lumen is associated with immune tolerance. In this setting, incoming *N. gonorrhoeae* is more likely to acquire tolerance (tolerance indicated by *N. gonorrhoeae* diplococci changing from red to green; DC—dendritic cell, IL—interleukin, PMN—polymorph neutrophil, TLR—toll-like receptor, PAMP—Pathogen-associated molecular pattern, figure produced in BioRender.com).

There are a number of possible explanations for the higher prevalence of AZM tolerance in anorectal than urethral infections. Urethral infections are typically symptomatic and of a short duration, whereas the vast majority of anorectal infections are asymptomatic and persist for months [20]. These differences are, in turn, related to factors such as differences in the microbiome and immune response in these locales (Figure 4). The rectal microbiome is considerably more diverse and abundant than the urethral microbiome [21,22]. A large number of bacterial species have been found to interact with *N. gonorrhoeae*. A number of bacterial species, such as numerous *Enterobacteriales* spp. that are prevalent in the anorectum, inhibit the growth of *N. gonorrhoeae* through the production of substances such as bacteriocins [23,24,25]. Various streptococcal and *Rothia* species have been noted to exhibit a similar effect [23,26]. It is possible that differences in these inhibitory effects between the urethra and anorectum may explain the higher prevalence of tolerance in the anorectum. Differences in the host immune responses between the urethra and anorectum may also play a role (Figure 4). The abundance of bacteria in the rectum is partially enabled by the downregulation of the host immune system at this site. For example, the toll-like receptors on the apical surface of the rectal epithelium are strongly downregulated [27]. The downregulated immune system in the rectum may favour the emergence of bacterial tolerance. A further possibility is that the longer duration of colonization in the anorectum than the urethra means that anorectal infections are more likely to be exposed to bystander selection from antibiotics used for other indications that, in turn, select for tolerance [28]. 

Our findings are commensurate with those of studies that have found that the anatomical site of infection selects for specific pheno- and genotypic traits in *N. gonorrhoeae*. One study, for example, found that the cervix selected for loss-of-function mutations in the *mtrCDE* and *farAB* efflux pumps, which were, in turn, associated with increased susceptibility to various antimicrobials [12]. Another study found that anorectal gonococcal infections exhibited a higher expression of the *mtrCDE* efflux pump than urethral infections [13]. The urethral isolates in our study had lower ciprofloxacin and azithromycin MICs than rectal isolates. Whilst some studies have found that MICs may vary by the site of infection [12], the majority of studies have not found this to be the case [29,30]. We cannot exclude the possibility that the higher azithromycin MICs in the anorectal isolates or some other unmeasured variable was responsible for the higher prevalence of AZM tolerance observed at this site. We attempted multivariable logistic regression to assess the relative impacts of the baseline azithromycin MIC and site of infection on azithromycin tolerance. These analyses were not possible due to the fact that there were no isolates with tolerance in the urethra, and thus, all the urethral isolates were dropped from the analysis (Supplementary Table S6).

The clinical cure rates for urethral and anorectal infections are typically high for most recommended treatments. This is less in the case of pharyngeal infections, where the cure rate for agents such as aminoglycosides and zoliflodacin is lower than for other sites [31]. Whilst poor drug penetration into the oropharynx likely plays an important role in this regard, it may be worthwhile testing the hypothesis that tolerance contributes to this poor cure rate. Studies in other species have found that tolerance plays a crucial role in the emergence of AMR [5,32]. Future studies will be required to assess if tolerance plays a similar role in *N. gonorrhoeae*.

It is possible that tolerance, just like resistance, could be underpinned by stochastic pheno- and genotypic variations. These variations could explain our finding of tolerance emerging in one or two of the three replicates. Only one of four published studies using TD tests reported conducting the test in replicate [6,7,33,34]. This one study reported conducting TD tests in duplicate but did not report if there was any discordance in the TD test between replicates [33]. Our study was thus the first to report discordant tolerance in the replicates. It is worth noting that using the replicates in the TD test, we identified discordant tolerance to both CIP and CRO in one WHO reference isolate (WHO-U). 

Previous studies have found tolerance in the clinical isolates of different bacterial species, such as methicillin-resistant *Staphylococcus aureus* (MRSA) blood infections, *Pseudomonas aeruginosa* infections in cystic fibrosis patients and *Enterococcus faecium* infection in a leukaemia patient [34,35,36]. Lazarovits et al. (2022) described how tolerance to multiple antibiotics, including ampicillin, CRO and ertapenem, was detected via the TD test in the *E. coli* isolates of patients with bloodstream infections. Importantly, they found that the detection of tolerance in *E. coli* was associated with an increased risk of reinfection [37]. 

The limitations of this study include the use of only TD tests to detect tolerance; other techniques, such as MDK99 killing curves, could have provided useful complementary information. In addition, no genotyping or transcriptomics was performed on the obtained tolerant colonies, as this was beyond the scope of the current study. However, we recently performed omics on tolerant colonies that will create a better understanding of the mutations associated with CRO tolerance in *N. gonorrhoeae* [38]. Although we tested all the clinical isolates in triplicate, we did not rerun the TD- tests on a separate occasion to assess the reproducibility of our findings. We only assessed if ceftriaxone-tolerant isolates could accelerate the emergence of ciprofloxacin resistance and did not include other antimicrobial combinations. Finally, we do not have an explanation for why there was no difference in the prevalence of ciprofloxacin tolerance between anatomical sites. 

Nonetheless, this is the first in vitro study to detect tolerance to AZM and CIP in clinical isolates of *N. gonorrhoeae*. This study established a difference in the prevalence of tolerance to AZM based on the infection site. Moreover, we used a large sample size (the biggest to date), performed the experiment in triplicate and performed the investigation blinded to infection sites. Future studies are required to confirm our finding of differences in tolerance by the site of infection (including the oropharynx) and to assess the clinical consequences (such as differences in infectivity) and epidemiological consequences (such as the probability of AMR emerging).

### Impact Statement

Tolerance, defined as the ability of a bacteria to survive transient antibiotic exposure without exhibiting a rise in the minimal inhibitory concentration (MIC), is a growing concern in high-priority pathogens, such as *N. gonorrhoeae*. This study confirmed the findings from a previous study using a larger sample size and three antimicrobials (ciprofloxacin, azithromycin and ceftriaxone) to detect tolerance in clinical and WHO reference isolates of *N. gonorrhoeae.* Furthermore, tolerance to these antibiotics varied significantly between anorectal and urogenital infection sites, with azithromycin tolerance particularly prominent in anorectal isolates.

## Figures and Tables

**Table 1:** Azithromycin, ciprofloxacin and ceftriaxone tolerance emergence following a TD test on 14 WHO *N. gonorrhoeae* reference strains.

ANTIBIOTICS	TOLERANCE	
	Number	WHO Isolates
AZITHROMYCIN	1	Z
CIPROFLOXACIN	1	P
CEFTRIAXONE	7	K, M, N, O, P, U, W

**Table 2:** Azithromycin, ciprofloxacin and ceftriaxone tolerance and potential tolerance emergence following a TD test on anorectal (*n* = 30) and urogenital (*n* = 32) *N. gonorrhoeae* clinical isolates.

ANTIBIOTICS	TOLERANCE			POTENTIAL TOLERANCE		
	Yes	No	Percent	Yes	No	Percent
AZITHROMYCIN						
ANORECTAL	4	26	13.3%	9	21	30%
UROGENITAL	0	32	0%	1	31	3.1%
	*p* value		0.033	*p* value		0.004
CIPROFLOXACIN						
ANORECTAL	2	28	6.7%	4	26	13.3%
UROGENITAL	2	30	6.3%	7	25	21.9%
	*p* value		0.947	*p* value		0.370
CEFTRIAXONE						
ANORECTAL	6	24	20%	10	30	33.3%
UROGENITAL	2	30	6.3%	2	30	6.3%
	*p* value		0.107	*p* value		0.007

**Table 3:** Association between azithromycin, ciprofloxacin and ceftriaxone tolerance following a TD test in all 62 *N. gonorrhoeae* clinical isolates [N (%)]. Fisher’s exact test.

Tolerance	Ceftriaxone		Ciprofloxacin	
	No	Yes	No	Yes
Azithromycin				
No	51 (88%)	7 (12%)	54 (93%)	4 (7%)
Yes	3 (75%)	1 (25%)	4 (100%)	0 (0%)
	*p*-value 0.433		*p*-value 1.0	
Ciprofloxacin				
No	52 (89%)	6 (10%)	-	-
Yes	2 (50%)	2 (50%)		
	*p*-value 0.077			

**Table 4:** Mixed-effects linear regression of association between tolerance and increase in *N. gonorrhoeae* ciprofloxacin MIC during cross-plating experiment, controlling for day of sampling (Day) and repeated measures of individual strains (Strain ID).

	COEF.	95% CI	*p*-VALUE
DAY	0.007	0.002–0.011	0.002
Tolerance	−0.004	−0.018–0.010	0.556
Random effects Strain id			
	1.85 × 10^−10^	2.7 × 10^−17^–0.001	

## Data Availability

Data is contained within the article.

## Supplementary Materials

The following supporting information can be downloaded at https://www.mdpi.com/article/10.3390/pathogens13070538/s1, Tables S1–S6: Table S1—Tolerance and Heterotolerance in WHO Reference Strains. Table S2—Tolerance to AZM, CIP and CRO in 62 isolates, and their site of infection. Table S3—MICs of the clinical isolates pre and post TD-test. Table S4—Increases in ciprofloxacin MIC by day during E-test cross plating. Table S5—Summary measures of MICs for ceftriaxone, azithromycin, and ciprofloxacin by site of infection. Table S6: Multivariable logistic regression to assess the relative impacts of the baseline azithromycin MIC and site of infection on azithromycin tolerance.

## References

[1] Brauner A., Fridman O., Gefen O., Balaban N.Q. (2016). Distinguishing between resistance, tolerance and persistence to antibiotic treatment. Nat. Rev. Microbiol..

[2] Sulaiman J.E., Lam H. (2021). Evolution of Bacterial Tolerance Under Antibiotic Treatment and Its Implications on the Development of Resistance. Front. Microbiol..

[3] Fridman O., Goldberg A., Ronin I., Shoresh N., Balaban N.Q. (2014). Optimization of lag time underlies antibiotic tolerance in evolved bacterial populations. Nature.

[4] Handwerger S., Tomasz A. (1985). Antibiotic Tolerance Among Clinical Isolates of Bacteria. Rev. Infect. Dis..

[5] Levin-Reisman I., Ronin I., Gefen O., Braniss I., Shoresh N., Balaban N.Q. (2017). Antibiotic tolerance facilitates the evolution of resistance. Science.

[6] Gefen O., Chekol B., Strahilevitz J., Balaban N.Q. (2017). TDtest: Easy detection of bacterial tolerance and persistence in clinical isolates by a modified disk-diffusion assay. Sci. Rep..

[7] Kotková H., Cabrnochová M., Lichá I., Tkadlec J., Fila L., Bartošová J., Melter O. (2019). Evaluation of TD test for analysis of persistence or tolerance in clinical isolates of *Staphylococcus aureus*. J. Microbiol. Methods.

[8] World Health Organisation (2016). WHO Guidelines for the Treatment of Neisseria Gonorrhoeae.

[9] Ohnishi M., Saika T., Hoshina S., Iwasaku K., Nakayama S.I., Watanabe H., Kitawaki J. (2011). Ceftriaxone-resistant *Neisseria gonorrhoeae*, Japan. Emerg. Infect. Dis..

[10] Unemo M., Golparian D., Nicholas R., Ohnishi M., Gallay A., Sednaoui P. (2012). High-level cefixime- and ceftriaxone-resistant *Neisseria gonorrhoeae* in France: Novel penA mosaic allele in a successful international clone causes treatment failure. Antimicrob. Agents Chemother..

[11] Balduck M., Laumen J.G.E., Abdellati S., De Baetselier I., de Block T., Manoharan-Basil S.S., Kenyon C. (2022). Tolerance to Ceftriaxone in *Neisseria gonorrhoeae*: Rapid Induction in WHO P Reference Strain and Detection in Clinical Isolates. Antibiotics.

[12] Ma K.C., Mortimer T.D., Hicks A.L., Wheeler N.E., Sánchez-Busó L., Golparian D., Taiaroa G., Rubin D.H., Wang Y., Williamson D.A. (2020). Adaptation to the cervical environment is associated with increased antibiotic susceptibility in *Neisseria gonorrhoeae*. Nat. Commun..

[13] Morse S.A., Lysko P.G., McFarland L., Knapp J.S., Sandstrom E., Critchlow C., Holmes K.K. (1982). Gonococcal strains from homosexual men have outer membranes with reduced permeability to hydrophobic molecules. Infect. Immun..

[14] Hauser C., Hirzberger L., Unemo M., Furrer H., Endimiani A. (2015). In vitro activity of fosfomycin alone and in combination with ceftriaxone or azithromycin against clinical *Neisseria gonorrhoeae* isolates. Antimicrob. Agents Chemother..

[15] Unemo M., Golparian D., Sánchez-Busó L., Grad Y., Jacobsson S., Ohnishi M., Lahra M.M., Limnios A., Sikora A.E., Wi T. (2016). The novel 2016 WHO *Neisseria gonorrhoeae* reference strains for global quality assurance of laboratory investigations: Phenotypic, genetic and reference genome characterization. J. Antimicrob. Chemother..

[16] McDermott P.F., White D.G., Zhao S., Simjee S., Walker R.D. (2008). Antimicrobial Susceptibility Testing. Preharvest and Postharvest Food Safety: Contemporary Issues and Future Directions.

[17] BioMérieux ETEST-Trusted Leader in MIC Gradient Strip Technology. https://www.biomerieux-usa.com/sites/subsidiary_us/files/prn_056750_rev_03.a_etest_brochure_final_art_2.pdf.

[18] Cirz R.T., Romesberg F.E. (2006). Induction and inhibition of ciprofloxacin resistance-conferring mutations in hypermutator bacteria. Antimicrob. Agents Chemother..

[19] Raisman J.C., Fiore M.A., Tomin L., Adjei J.K., Aswad V.X., Chu J., Domondon C.J., Donahue B.A., Masciotti C.A., McGrath C.G. (2022). Evolutionary paths to macrolide resistance in a Neisseria commensal converge on ribosomal genes through short sequence duplications. PLoS ONE.

[20] Chow E.P., Camilleri S., Ward C., Huffam S., Chen M.Y., Bradshaw C.S., Fairley C.K. (2016). Duration of gonorrhoea and chlamydia infection at the pharynx and rectum among men who have sex with men: A systematic review. Sex. Health.

[21] Dekaboruah E., Suryavanshi M.V., Chettri D., Verma A.K. (2020). Human microbiome: An academic update on human body site specific surveillance and its possible role. Arch. Microbiol..

[22] Galiwango R.M., Park D.E., Huibner S., Onos A., Aziz M., Roach K., Anok A., Nnamutete J., Isabirye Y., Wasswa J.B. (2022). Immune milieu and microbiome of the distal urethra in Ugandan men: Impact of penile circumcision and implications for HIV susceptibility. Microbiome.

[23] Akomoneh E.A., Laumen J.G.E., Abdellati S., Van Dijck C., Vanbaelen T., Britto X.B., Manoharan-Basil S.S., Kenyon C. (2022). The Discovery of Oropharyngeal Microbiota with Inhibitory Activity against Pathogenic *Neisseria gonorrhoeae* and Neisseria meningitidis: An In Vitro Study of Clinical Isolates. Microorganisms.

[24] Baquero F., Moreno F. (1984). The microcins. FEMS Microbiol. Lett..

[25] Simpson D.M., Davis C.P. (1979). Properties of a gonococcal inhibitor produced by *Escherichia coli*. J. Gen. Microbiol..

[26] McBride M.E., Duncan W.C., Knox J.M. (1980). Bacterial interference of *Neisseria gonorrhoeae* by alpha-haemolytic streptococci. Br. J. Vener. Dis..

[27] Yu L.C.-H., Wang J.-T., Shu-Chen W., Ni Y.-H. (2012). Host-microbial interactions and regulation of intestinal epithelial barrier function: From physiology to pathology. World J. Gastrointest. Pathophysiol..

[28] Tedijanto C., Olesen S.W., Grad Y.H., Lipsitch M. (2018). Estimating the proportion of bystander selection for antibiotic resistance among potentially pathogenic bacterial flora. Proc. Natl. Acad. Sci. USA.

[29] Kidd S., Zaidi A., Asbel L., Baldwin T., Gratzer B., Guerry S., Kerani R.P., Pathela P., Pettus K., Soge O.O. (2015). Comparison of antimicrobial susceptibilities of pharyngeal, rectal, and urethral *Neisseria gonorrhoeae* isolates among men who have sex with men. Antimicrob. Agents Chemother..

[30] Jacobsson S., Cole M.J., Spiteri G., Day M., Unemo M. (2021). Associations between antimicrobial susceptibility/resistance of *Neisseria gonorrhoeae* isolates in European Union/European Economic Area and gender, sexual orientation and anatomical site of infection, 2009–2016. BMC Infect. Dis..

[31] Kong F.Y., Hatzis C.L., Lau A., Williamson D.A., Chow E.P., Fairley C.K., Hocking J.S. (2020). Treatment efficacy for pharyngeal *Neisseria gonorrhoeae*: A systematic review and meta-analysis of randomized controlled trials. J. Antimicrob. Chemother..

[32] Levin-Reisman I., Brauner A., Ronin I., Balaban N.Q. (2019). Epistasis between antibiotic tolerance, persistence, and resistance mutations. Proc. Natl. Acad. Sci. USA.

[33] Khan M., Ma K., Wan I., Willcox M.D. (2023). Ciprofloxacin resistance and tolerance of Pseudomonas aeruginosa ocular isolates. Contact Lens Anterior Eye.

[34] Liu J., Gefen O., Ronin I., Bar-Meir M., Balaban N.Q. (2020). Effect of tolerance on the evolution of antibiotic resistance under drug combinations. Science.

[35] Mulcahy L.R., Burns J.L., Lory S., Lewis K. (2010). Emergence of *Pseudomonas aeruginosa* Strains Producing High Levels of Persister Cells in Patients with Cystic Fibrosis. J. Bacteriol..

[36] Honsa E.S., Cooper V.S., Mhaissen M.N., Frank M., Shaker J., Iverson A., Rubnitz J., Hayden R.T., Lee R.E., Rock C.O. (2017). RelA Mutant Enterococcus faecium with Multiantibiotic Tolerance Arising in an Immunocompromised Host. MBio.

[37] Lazarovits G., Gefen O., Cahanian N., Adler K., Fluss R., Levin-Reisman I., Ronin I., Motro Y., Moran-Gilad J., Balaban N.Q. (2022). Prevalence of Antibiotic Tolerance and Risk for Reinfection Among *Escherichia coli* Bloodstream Isolates: A Prospective Cohort Study. Clin. Infect. Dis..

[38] Manoharan-Basil S., Balduck M., Laumen J., Kenyon C. Transcriptomic profiling of ceftriaxone-tolerant phenotypes of *Neisseria gonorrhoeae* WHO P reference strain. Proceedings of the ECCMID.

